# Evidence for a Role of the Long Non-Coding RNA ITGB2-AS1 in Eosinophil Differentiation and Functions

**DOI:** 10.3390/cells13231936

**Published:** 2024-11-22

**Authors:** Timothée Fettrelet, Aref Hosseini, Jacqueline Wyss, Joanna Boros-Majewska, Darko Stojkov, Shida Yousefi, Hans-Uwe Simon

**Affiliations:** 1 Institute of Pharmacology, University of Bern, 3010 Bern, Switzerland; 2 Department of Visceral Surgery and Medicine, Inselspital, Bern University Hospital, University of Bern, 3010 Bern, Switzerland; 3 Institute of Biochemistry, Brandenburg Medical School, 16816 Neuruppin, Germany

**Keywords:** differentiation, degranulation, eosinophil, eosinophil peroxidase, HL-60, ITGB2-AS1, long non-coding RNA, reactive oxygen species

## Abstract

Eosinophils, a type of granulocyte derived from myeloid precursors in the bone marrow, are distinguished by their cytoplasmic granules. They play crucial roles in immunoregulation, tissue homeostasis, and host defense, while also contributing to the pathogenesis of various inflammatory diseases. Although long non-coding RNAs (lncRNAs) are known to be involved in eosinophilic conditions, their specific expression and functions within eosinophils have not been thoroughly investigated, largely due to the reliance on tissue homogenates. In an effort to address this gap, we analyzed publicly available high-throughput RNA sequencing data to identify lncRNAs associated with eosinophilic conditions. Among the identified lncRNAs, ITGB2 antisense RNA 1 (*ITGB2-AS1*) was significantly downregulated in blood eosinophils from patients with hypereosinophilia. To further explore its role in eosinophil biology, we generated a stable *ITGB2-AS1* knockdown in the HL-60 cell line. Interestingly, *ITGB2-AS1* deficiency led to impaired eosinophil differentiation, as evidenced by a reduction in cytoplasmic granules and decreased expression of key eosinophil granule proteins, including eosinophil peroxidase (EPX) and major basic protein-1 (MBP-1). Additionally, *ITGB2-AS1*-deficient cells exhibited compromised eosinophil effector functions, with reduced degranulation and impaired production of reactive oxygen species (ROS). These findings suggest that *ITGB2-AS1* plays a pivotal role in eosinophil differentiation and function, positioning it as a novel regulator in eosinophil biology.

## 1. Introduction

Eosinophils are a subtype of granulocytes that are evolutionarily conserved across vertebrates [1,2]. These bone marrow-derived cells originate from hematopoietic stem cells (HSCs) and undergo a series of developmental stages, progressing through common myeloid progenitors (CMPs), eosinophil progenitors (EoPs), myeloblasts (MBs), promyelocytes (pMCs), and myelocytes (MCs), before differentiating into mature eosinophils [3]. The process of eosinopoiesis is regulated by transcription factors, including GATA-1, GATA-2, PU.1, C/EBPs, IRF8, FOG-1, and XBP1, and is promoted by the cytokines granulocyte-macrophage colony-stimulating factor (GM-CSF), interleukin-3 (IL-3), and interleukin-5 (IL-5) [4]. The formation of granules during eosinophil differentiation begins with the development of primary granules, also referred to as early secondary granules, in promyelocytes [5]. Subsequently, the myelocyte stage is marked by the emergence of specific granules, also referred to as crystalloid or secondary granules, which are predominantly found in mature eosinophils [6]. These granules facilitate the storage of four preformed toxic cationic proteins: eosinophil peroxidase (EPX), major basic protein-1 (MBP-1), eosinophil cationic protein (ECP), and eosinophil-derived neurotoxin (EDN), along with a broad range of cytokines, chemokines, and growth factors [4,7,8].

Mature eosinophils represent a minority of 1–5% of total circulating leukocytes in human peripheral blood with an absolute eosinophil count (AEC) generally below 500 cells/μL under normal conditions [9]. Upon entry into the circulation, mature eosinophils are recruited to tissues, such as the lungs, thymus, mammary glands, uterus, and non-esophageal parts of the gastrointestinal tract, in response to eotaxins and other chemokines [1,10,11]. Although eosinophils have a short half-life of only a few hours in circulation, they can prolong their survival in tissues through the support of cytokines [12]. Traditionally regarded as cytotoxic effector cells, eosinophils are now widely recognized for their broader immunomodulatory and homeostatic roles [13]. They participate in host defense against bacteria, helminths, parasites, and viruses, while also contributing to the pathology of various allergic and non-allergic inflammatory diseases [4]. Eosinophilic disorders, which include gastrointestinal and lung disorders, hypereosinophilic syndromes (HESs), dermatoses, allergic and infectious diseases, drug responses, and neoplastic disorders, are typically characterized by eosinophil-rich inflammatory infiltrates or extracellular deposition of eosinophil-derived proteins, which may result in local inflammation, tissue damage, remodeling, and organ pathology [4,9,14,15]. However, their precise function in both health and disease remains incompletely understood and continues to be a subject of ongoing research and discussion.

Long non-coding RNAs (lncRNAs) are a class of regulatory RNAs involved in a wide range of cellular processes, typically defined as non-protein-coding transcripts exceeding 200 nucleotides in length [16]. In 2007, Wagner et al. demonstrated that the lncRNA eosinophil granule ontogeny transcript (EGOT) was expressed during eosinophil development and crucial for the expression of the granule proteins MBP-1 and EDN [17]. Additionally, numerous studies examined the role of lncRNAs in eosinophil-related conditions, such as asthma [18,19], allergic rhinitis [20], and eosinophilic esophagitis (EoE) [21]. For instance, elevated levels of the lncRNA RP11-401.2 were detected in blood samples from bronchial asthma patients [18]. Similarly, the lncRNA LNC_000127 was found to be upregulated in the blood of eosinophilic asthma patients and shown to play a role in the regulation of Th2 inflammatory responses [19]. Furthermore, increased expression of BRAF-activated non-coding RNA (BANCR) was observed in esophageal biopsies from patients with active EoE and to be inducible in primary esophageal epithelial cells treated with IL-13 [21]. However, these studies mainly focused on assessing RNA expression levels in tissue homogenates, neglecting the specific expression and functions of lncRNAs in eosinophils, and leaving their potential role in the pathogenesis of related disorders largely unexplored.

This study aimed to identify relevant lncRNA candidates associated with eosinophil biology using computational analyses and to further characterize their role in eosinophil differentiation and function. Through the analysis of publicly available high-throughput RNA sequencing (RNA-seq) datasets, we identified eight promising lncRNA candidates linked to eosinophil-related disorders. We then confirmed the expression of these lncRNAs in human eosinophils from both healthy donors and patients with hypereosinophilia (HE), which revealed a significant downregulation of *ITGB2-AS1* in HE patients.

To further investigate the role of *ITGB2-AS1* in eosinophil biology, we generated a stable knockdown of this lncRNA in the promyelocytic leukemia cell line HL-60 clone 15 (HL-60c15). Notably, *ITGB2-AS1*-deficient HL-60c15 cells exhibited impaired eosinophil differentiation, characterized by a significant reduction in cytoplasmic specific granules and decreased expression of eosinophil granule proteins, including EPX and MBP-1. Additionally, the *ITGB2-AS1* knockdown cells displayed compromised eosinophil effector functions, such as reduced degranulation and impaired production of reactive oxygen species (ROS), underscoring the critical role of *ITGB2-AS1* in eosinophil cytotoxic activity.

Collectively, our findings suggest that *ITGB2-AS1* is a novel and essential regulator of eosinophil differentiation and function.

## 2. Materials and Methods

### 2.1. Reagents

The reagents and their supplier information are provided in Table 1 below, organized alphabetically.

### 2.2. Identification of lncRNAs Upregulated in Human Blood Eosinophils

A manual query of the Haemopedia Human RNA-Seq database [22], hosting transcriptomic data of circulating white blood cells (WBCs), was performed. Genes significantly upregulated in eosinophils compared with other WBCs, including neutrophils, monocytes, dendritic cells (DCs), B cells, T cells, and natural killer (NK) cells, were selected and filtered for non-coding transcripts of more than 200 bp annotated as or spanning a region annotated as lncRNA by GENCODE annotation.

### 2.3. Identification of Eosinophil-Related Proteins

Genes associated with eosinophil maturation, regulation, and functions were selected from the literature and incorporated into the analysis (Table S1). These genes were grouped into four main categories. The first category included regulators of eosinophil maturation, which comprised well-established factors influencing eosinophil differentiation as previously described [23,24]. The second category focused on surface molecules expressed by eosinophils, for which the expression of consensus-listed molecules was verified through primary sources with experimental validation by flow cytometry [25,26]. The third category consisted of genes coding for secretory mediators [27,28,29]. The final category included genes coding for granule proteins and other proteins [7,29].

### 2.4. Selection of Datasets of Eosinophil-Related Diseases and RNA-Sequencing Analysis

A query of the GEO database identified 39 datasets from 35 studies using the following keywords: “eosinophil, eosinophilic, atopic, asthma, allergy, allergic, hypereosinophil*, DRESS, polyangiitis, esophagitis, rhinitis, dermatitis”. After filtering, we retained datasets that met the following criteria: inclusion of eosinophils in the studied tissue, establishment as case-control studies, and adequate sample sizes for normalization. The raw sequencing files of all the selected datasets were retrieved from the SRA database using the SRA toolkit, applying either the single-end or paired-end option based on the SRA metadata. The quality of the RNA-seq data was assessed using FastQC. Adapter trimming and low-quality read removal were performed using Trimmomatic with standard options. The reads were subsequently mapped to the reference genome (Homo_sapiens.GRCh38.84) using HISAT2 with default settings and prebuilt index files [30]. FeatureCounts from the R package Rsubread was used to count the number of reads overlapping with each gene, as specified in the genome annotation (Homo_sapiens.GRCh38.84), with the “transcript” option selected and excluding multi-overlaps [31]. Subsequent data normalization and differential gene expression analysis were performed using DESeq2 [32].

### 2.5. Correlation Network Analysis

The correlation between the RNA expression of all pre-selected lncRNAs and eosinophil-related protein-coding genes was assessed using the “rcorr” function from the R package Hmisc, yielding correlation coefficients (r) and their corresponding *p*-values. We supplied the transcript count matrix and applied Pearson correlation analysis, resulting in the generation of corresponding *p*-values. These *p*-values were then adjusted for multiple testing using the Benjamini–Hochberg procedure via the “p.adjust” function from the Stats R package. We further refined the final list by filtering significant correlations, specifically only those with adjusted *p*-values < 0.05 and a correlation coefficient (r) greater than 0.7. A connection in the correlation network was then defined as any correlation between a lncRNA and an eosinophil-related protein-coding gene meeting these criteria.

To visualize the correlations between RNA levels of lncRNAs and eosinophil-related protein-coding genes, the correlation networks were imported into the Cytoscape application. The most relevant lncRNAs for further characterization were selected based on a visual assessment of network clustering around protein-coding genes of interest, as well as the strength and number of connections to eosinophil-related protein-coding genes (minimum of 4 connections).

### 2.6. Purification of Human Eosinophils

Human eosinophils were purified from the peripheral blood of healthy individuals and (hyper)eosinophilic patients, as previously described [33,34]. Briefly, WBCs were separated by density-gradient centrifugation (800× *g*, 20 min, room temperature (RT)) using Pancoll Human (density of 1.077 g/mL) (PAN-Biotech). The erythrocytes in the granulocyte fraction were lysed with a lysis solution (10 mM KHCO_3_, 155 mM NH_4_Cl). Eosinophils were isolated from the granulocyte fraction devoid of erythrocytes by negative selection using the EasySep Human Eosinophil Isolation Kit (StemCell Technologies). The purity of purified eosinophils was ≥95% as assessed by the Hemacolor Rapid staining kit (Merck Millipore) followed by light microscopic analysis.

This study received approval from the Ethics Committee of the Canton of Bern, and written informed consent was obtained from the different blood donors.

### 2.7. Generation and Culture of Stably Transduced HL-60 Clone 15 Cells

The human promyelocytic leukemia cell line HL-60 clone 15 cells (HL-60c15, ATCC CRL-1964) was purchased from ATCC (Manassas, VA, USA). HL-60c15 cells were maintained in RPMI-1640/GlutaMAX medium supplemented with 10% FCS, 100 U/mL penicillin, and 100 µg/mL streptomycin at 37 °C in 5% CO_2_. HL-60c15 were differentiated into eosinophil-like cells (ELCs) in the presence of 20% FCS, 0.5 mM sodium butyrate, and 10 ng/mL of IL-5 for a maximum of 6 days. Lentiviral constructs (pCLenti-U6-shRNA-CMV-Puro-WPRE) coding for shRNA targeting the lncRNA *ITGB2-AS1* (shITGB2-AS1), as well as shRNA negative control (shControl), were purchased from OBiO Technology (Shanghai, China) (Table S2). Lentiviral particles were produced by transfecting the shRNA constructs together with the lentiviral envelope plasmid (PMD2.G) and the lentiviral packaging plasmid (psPAX2) (both provided by Dr. D. Trono) in HEK-293T cells with X-tremeGENE™ HP DNA Transfection Reagent (Roche Diagnostics). Lentiviral particles were then freshly used to transduce HL-60c15 cells in the presence of 8 µg/mL polybrene. Two days later, transduced cells were selected by the addition of 1 µg/mL puromycin to the cell culture medium for 3 weeks.

### 2.8. HL-60c15 Differentiation into Eosinophil-like Cells

HL-60c15 were differentiated into ELCs in RPMI-1640/GlutaMAX medium supplemented with 20% FCS, 0.5 mM sodium butyrate [35], and 10 ng/mL IL-5 for a maximum of 6 days.

#### 2.8.1. Cell Granularity

During differentiation, the granularity of ELCs was assessed manually by light microscopic analysis on a Leica DME microscope (Leica, Wetzlar, Germany) with a C plan 100×/1.25 Oil objective following Hemacolor Rapid staining (Merck Millipore). In addition, cell granularity was evaluated by measuring the side scatter of ELCs by flow cytometry (FACSLyric, BD Biosciences) and analyzed using FlowJo 10.5.3 software (Tree Star, Ashland, OR, USA).

#### 2.8.2. Flow Cytometry

The differentiation of ECLs was assessed by surface staining. Briefly, single-cell suspensions (0.3 × 10^6^/50 µL) were washed with 1 mL of washing buffer (PBS with 2% FCS) and incubated for 10 min in blocking buffer (PBS with 10% FCS and 10% IVIG) to block the Fc receptors. Dead cells were stained with BD Horizon™ Fixable Viability Stain 620 (1:200 dilution) for 35 min on ice.

Simultaneously, surface staining was performed with three monoclonal antibody panels, each incubated for 35 min on ice. In the first panel, the surface expression of CD11b and CCR3 was assessed with the following antibodies: APC anti-mouse/human CD11b (1:400 dilution) and Brilliant Violet 421™ anti-human CD193 (CCR3) (1:50 dilution). Following the surface staining with the first panel, cells were further stained for intracellular Ki-67 detection (Section 2.9.1). In the second panel, the surface expression of CCR1, CXCR3, and CD18 was determined with the following antibodies: Alexa Fluor^®^ 647 anti-human CD191 (CCR1) (1:20 dilution), Alexa Fluor^®^ 488 anti-human CD183 (CXCR3) (1:20 dilution), and PE anti-human CD18 (ITGB2) (1:5 dilution). The third panel assessed the surface expression of CD48 and CD52 with the following antibodies: Brilliant Violet 421™ anti-human CD48 (1:20 dilution) and PE anti-human CD52 (1:20 dilution). Samples were acquired by flow cytometry (FACSLyric, BD Biosciences) and analyzed using FlowJo 10.5.3 software (Tree Star, Ashland, OR, USA).

### 2.9. Cell Proliferation

#### 2.9.1. Flow Cytometry

Following the surface staining with the first antibody panel (chapter 2.8.2), the cells were fixed in 2% paraformaldehyde (PFA) for 10 min and subsequently permeabilized with 0.5% saponin in blocking buffer (PBS with 10% FCS and 10% IVIG). For intracellular Ki-67 detection, the cells were incubated with Alexa Fluor^®^ 488 anti-Human Ki-67 (1:20) for 30 min at RT, washed with PBS, and fixed in 1% PFA. Samples were acquired by flow cytometry (FACSLyric, BD Biosciences) and analyzed using FlowJo 10.5.3 software (Tree Star, Ashland, OR, USA).

#### 2.9.2. Absolute Cell Count

The proliferative status of differentiating HL-60c15 cells was evaluated by absolute cell count every 2 days of the differentiation for 6 days using the automated hematology analyzer Sysmex XP-300 (Sysmex Digitana, Horgen, Switzerland).

### 2.10. Confocal Laser Scanning Microscopy

Confocal microscopy analysis of differentiating HL-60c15 cells was performed as previously described [33,34]. Briefly, differentiating HL-60c15 cells (0.4 × 10^6^/100 µL X-VIVO 15 medium) were seeded on 12 mm glass coverslips and incubated for 30 min at 37 °C in 5% CO_2_. The cells were then fixed with 4% PFA for 5 min, washed with PBS (pH 7.4), and permeabilized with 0.05% saponin in PBS (pH 7.4) for 3 min at RT. Thereafter, the immunofluorescence staining was performed in the presence of 0.01% saponin. Non-specific binding was prevented by incubation of cells in blocking buffer (comprising human immunoglobulins, secondary antibody species serum, and 7.5% BSA) at RT for 10 min. Primary monoclonal mouse anti-mouse EPX antibody (1:400 dilution) and monoclonal mouse anti-human PRG2 antibody (1:100 dilution) were diluted in blocking solution and incubated with the cells at RT for 2 h. Subsequently, the secondary antibody goat anti-mouse Alexa Fluor 488 (1:400 dilution) was incubated at RT for 1 h. For controls, cells were stained with secondary antibodies only. Cells were subsequently washed in PBS (pH 7.4), stained with Hoechst 33342 (1 µg/mL) for 10 min, and mounted with Prolong Gold Antifade mounting medium.

Images were acquired using a confocal laser scanning microscope LSM 800 (Carl Zeiss Micro Imaging, Jena, Germany) with a Plan-Apochromat 40×/1.4 Oil DIC objective. The mean fluorescence intensity (MFI) of EPX (green channel) and MBP-1 (green channel represented in red in the images) was quantified within the cells, which were delineated using the “Surfaces” mode in Imaris 10.0.1 software (Bitplane AG, Zurich, Switzerland). To optimize the image display, min/max thresholds and gamma correction were used by Imaris 10.0.1 software (Bitplane AG, Zurich, Switzerland).

### 2.11. Immunoblotting

Immunoblotting was performed as previously described [36,37]. Briefly, cell lysates were prepared by resuspending the cell pellets in lysis buffer (50 mM Tris (pH 7.4), 150 mM NaCl, 10% glycerol, 1% Triton X-100, 1% NP-40, 2 mM EDTA, 2.5 mM MgCl_2_, 2.5 mM NaF, 10 mM NaPyrophosphate, and 200 μM Na_3_VO_4_) freshly supplemented with protease inhibitor cocktail (Sigma-Aldrich), 1 mM PMSF (Sigma-Aldrich), 5.7 mM diisopropyl fluorophosphate (DFP) (Sigma-Aldrich), and 1× PhosSTOP™ phosphatase inhibitor cocktail (Roche). Cells were collected, washed with PBS, and lysed on ice for 20 min. Protein lysates were then collected after high-speed centrifugation (13,000 rpm, 15 min, 4 °C). The protein concentration for each sample was quantified with a Pierce BCA protein assay kit (Thermo Fisher Scientific). Extracted proteins (50 μg) were denatured and separated on 12% SERVAGel TG PRiME gels (SERVA Electrophoresis, Heidelberg, Germany), followed by protein transfer onto Immobilon-P PVDF membrane (Merck Millipore). Membranes were blocked in 5% non-fat milk in TBST (0.1% Tween 20 in 20 mM Tris and 150 mM NaCl [pH 7.6]) for 1 h and incubated with primary antibodies at 4 °C overnight. The antibodies used for immunoblotting were monoclonal mouse anti-mouse EPX antibody (1:1000 dilution) or monoclonal mouse anti-human GAPDH antibody (1:2000 dilution). Horseradish peroxidase (HRP)-coupled sheep anti-mouse secondary antibodies (1:5000 dilution) were added to the membrane for 1 h at RT. After washing three times the membranes with TBST, the signal was detected by chemiluminescence using the Immobilon Forte Western HRP substrate (Merck Millipore). Images were acquired on the Odyssey Fc Imaging System (LI-COR Biosciences, Lincoln, NE, USA) and analyzed with Image Studio 3.1.4 software (LI-COR Biosciences).

### 2.12. Quantitative RT-qPCR

Cells were washed in cold PBS supplemented with RNasin^®^ Plus Ribonuclease Inhibitor prior to cell lysis, as previously described [36]. RNA was then extracted from ELCs differentiated from HL-60c15 cell line using TRIzol™ Reagent (Invitrogen) and from human circulating blood eosinophils using Quick-RNA™ Microprep Kit (ZymoResearch), respectively. Subsequently, cDNA was synthesized from 2000 ng of RNA (human eosinophils) or 1000 ng of RNA (HL-60c15) using the iScript™ gDNA Clear cDNA Synthesis kit (Bio-Rad Laboratories), following the manufacturer’s instructions. Quantitative PCR (qPCR) was performed using the iTaq Universal SYBR Green Supermix (Bio-Rad Laboratories) with the CFX Connect Real-Time PCR Detection system (Bio-Rad Laboratories). The primers used in this study (Table S3) were synthesized by Microsynth AG (Balgach, Switzerland). The target gene RNA levels were analyzed using the ΔΔCt method, normalized to the geometric mean of the housekeeping genes GAPDH (human), UBC (human), or HPRT1 (human), and represented relative to control samples or undifferentiated shControl cells. The amplification protocol was as follows: 40 cycles (95 °C for 10 s, 60 °C for 20 s, 72 °C for 15 s).

### 2.13. Degranulation Assays

#### 2.13.1. EPX Release

The colorimetric detection of eosinophil peroxidase (EPX) in the supernatants of stimulated ELCs was adapted from a previously reported method for human eosinophils [33,38]. Briefly, HL-60c15-derived ELCs after 6 days of differentiation (in the presence of 20% FCS, 0.5 mM sodium butyrate, and 10 ng/mL IL-5) were resuspended (0.15 × 10^6^/150 µL X-VIVO 15 medium). The cells were primed with 25 ng/mL GM-CSF or IL-3. Cytochalasin B (5 µM) was added to the cell suspension in the last 5 min of priming. Cytokine-primed cells were subsequently stimulated with 10 nM C5a. After 30 min of stimulation, supernatants were collected following centrifugation (5 min, 1400 rpm, 4 °C), and the release of EPX was assessed by adding 150 µL of O-phenylenediamine (OPD) substrate to the supernatants for 2 min at RT. The substrate solution was freshly prepared by adding 800 µL of 5 mM OPD to 4 mL 1 M Tris (pH 8.0), 1.25 µL 30% H_2_O_2,_ and 5.2 mL distilled water. The mix of supernatant and substrate was then transferred into a black, glass-bottom 96-well plate, and the absorbance was measured at 492 nm with a spectrofluorometer SpectraMax M2 plate reader (Molecular Devices, Biberach an der Riss, Germany).

#### 2.13.2. Surface Expression of the Surrogate Marker CD63

HL-60c15-derived ELC degranulation was evaluated by increased surface expression of the surrogate marker CD63, based on a previously reported method on human eosinophils [33,36,39]. Briefly, HL-60c15-derived ELCs after 6 days of differentiation (in the presence of 20% FCS, 0.5 mM sodium butyrate, and 10 ng/mL IL-5) were resuspended in X-VIVO 15 medium (0.5 × 10^6^/200 µL). The cells were primed with 25 ng/mL GM-CSF or IL-3. Cytochalasin B (5 µM) was added to the cell suspension in the last 5 min of priming. Cytokine-primed cells were subsequently stimulated with 10 nM C5a. After 30 min of stimulation, the cells were washed with 1 mL of washing buffer (PBS with 2% FCS) and incubated for 10 min in blocking buffer (PBS with 10% FCS and 10% IVIG) to block the Fc receptors. Dead cells were stained with BD Horizon™ Fixable Viability Stain 620 (Cat. #564996, BD Biosciences, Allschwil, Switzerland; 1:200 dilution) for 30 min on ice. Simultaneously, CD63 protein levels on the cell surface were determined using APC-conjugated anti-human CD63 antibody (clone H5C6; Cat. #353008; BioLegend, London, UK; 1:50 dilution) incubated for 30 min on ice. Samples were acquired by flow cytometry (FACSLyric, BD Biosciences) and analyzed using FlowJo 10.5.3 software (Tree Star, Ashland, OR, USA).

### 2.14. Reactive Oxygen Species (ROS) Measurements

The method to analyze ROS production from HL-60c15-derived ELCs was adapted from previous studies [34,40]. Briefly, following 6 days of differentiation in RPMI-1640/GlutaMAX medium supplemented with 20% FCS, 0.5 mM sodium butyrate, and 10 ng/mL IL-5, HL-60c15-derived eosinophils were resuspended in RPMI-1640/GlutaMAX medium (0.5 × 10^6^/200 µL) supplemented with 5% FCS. The cells were primed with 25 ng/mL GM-CSF or IL-3. In the last 10 min of priming, 1 µM DHR123 was added to the cells. Cytokine-primed cells were subsequently stimulated with 10 nM C5a for 30 min at 37 °C. A total of 100 µL of the cell suspension was finally transferred to black, glass-bottom 96-well plates (Greiner Bio-One) in duplicates. ROS activity was measured using a spectrofluorometer (SpectraMax M2, Molecular Devices).

### 2.15. Statistical Analysis

Statistical analysis of all the data was performed using GraphPad Prism 8 software (GraphPad Software Inc., La Jolla, CA, USA). Data are represented as the mean values ± SEM. The numbers of independent replicates for each experiment are reported in the figure legends. To compare groups, the Mann-Whitney test or two-way ANOVA with Tukey’s multiple comparisons test were applied, and *p*-values < 0.05 were considered statistically significant.

## 3. Results

### 3.1. Co-Expression Analysis of Upregulated incRNAs in Eosinophils and Eosinophil-Related Protein-Coding Genes in Transcriptomic Datasets of Eosinophil-Associated Diseases Identifies Eight Promising incRNA Candidates

**Figure 1:**
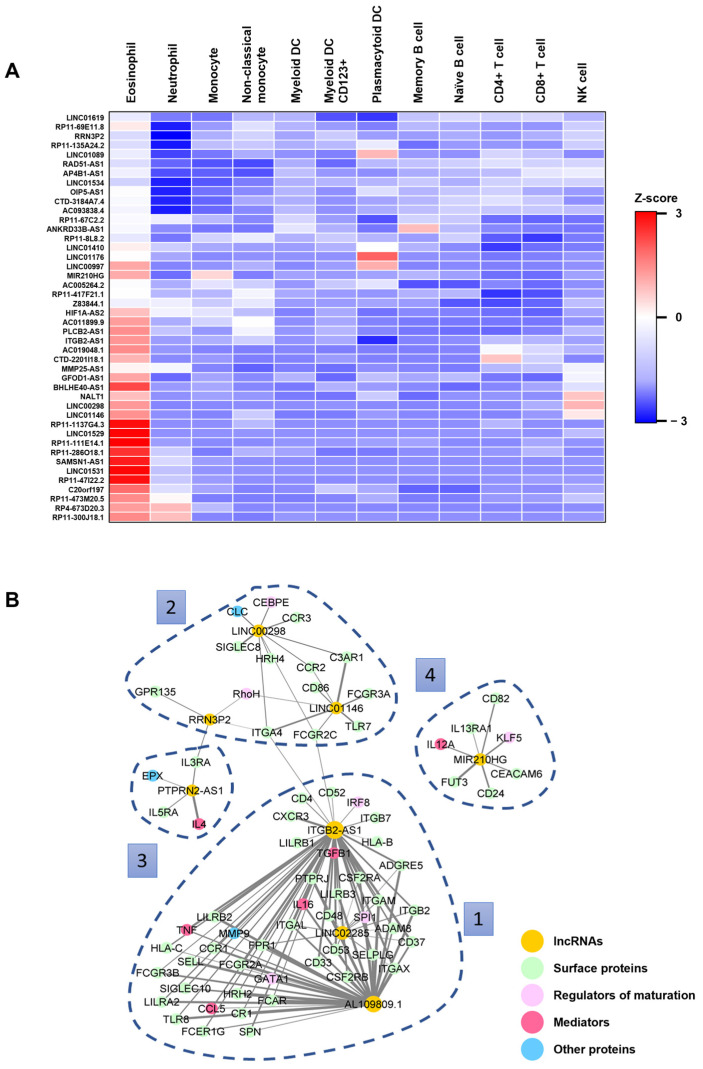
Identification of eight lncRNA candidates predicted *in silico* from human transcriptomic datasets of eosinophil-related diseases. (**A**) Identification of lncRNAs. Heatmap illustrating 44 lncRNAs that are differentially expressed in eosinophils compared with other WBCs, identified through a manual query of the Haemopedia dataset [22]. The color key represents row Z-scores, with red indicating overexpression and blue representing downregulation. (**B**) Correlation network analysis. Correlation network between *in silico* predicted lncRNAs (yellow) and a gene list of eosinophil-related proteins (color-coded by protein type). Edges represent significant correlations (*p*-values < 0.05) with a correlation coefficient greater than 0.7. Four distinct clusters were manually defined based on the network of eosinophil-related protein-coding genes associated with the different lncRNAs. Abbreviations: lncRNA, long non-coding RNA; WBC, white blood cell.

To identify potential key lncRNAs implicated in eosinophil biology, we initially conducted a manual search within the publicly available Haemopedia RNA-seq database, housing transcriptional profiles of human peripheral blood leukocytes [22]. LncRNAs exhibiting significant upregulation in human eosinophils compared with other white blood cell (WBC) types were selected, resulting in a curated list of 44 promising lncRNAs (Figure 1A). Concurrently, a compilation of genes associated with eosinophil biology (Table S1) was derived from pertinent literature, encompassing regulators of eosinophil differentiation [23,24], eosinophil surface markers [25,26], secretory mediators [27,28,29], and granule proteins [7,29]. Subsequently, we conducted a correlation analysis between the identified lncRNAs (Figure 1A) and the pool of eosinophil-associated protein-coding genes (Table S1) using transcriptomic datasets associated with eosinophil-related diseases (Table 2), such as allergic rhinitis, bronchial asthma, EoE, atopic dermatitis (AD), and eosinophilic granulomatosis with polyangiitis (EGPA). The resulting Pearson correlations were refined to display only those that were statistically significant (Benjamini–Hochberg adjusted *p*-values < 0.05) and exhibited a strong association (correlation coefficient, r > 0.7 (Figure 1B).

We observed a correlation between the expression levels of eight lncRNAs upregulated in eosinophils and various eosinophil-related protein-coding genes. The remaining 36 upregulated lncRNAs in eosinophils (Figure 1A), which did not exhibit a significant correlation with the protein-coding genes, were excluded from the network (Figure 1B). Among the lncRNAs in the network, *LINC01146* has been previously reported to exert both positive and negative effects on the growth and metastasis of different cancer types [41,42]. Similarly, the lncRNAs *MIR210HG* and *ITGB2-AS1* have been shown to promote the progression of various cancer types [43,44,45]. Interestingly, the potential functions of the remaining lncRNAs, namely *RRN3P2*, *PTPRN2-AS1*, *AL109809.1*, *LINC02285*, and *LINC00298*, have not been well characterized yet.

### 3.2. The lncRNA ITGB2-AS1 Is Expressed in Human Eosinophils and Exhibits Marked Downregulation in Blood Eosinophils from Patients with Hypereosinophilia

**Figure 2:**
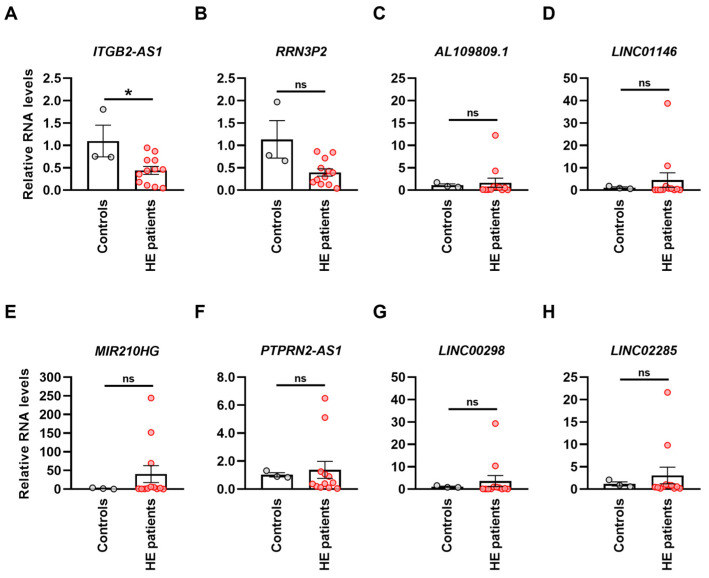
Abundance of *in silico*-predicted lncRNAs in blood eosinophils from healthy controls and hypereosinophilic patients. (**A**–**H**) Quantitative PCR. Abundance of the lncRNAs identified through correlation network analysis of eosinophil-related disease datasets in circulating eosinophils from the blood of healthy controls and HE patients. The name of each lncRNA is indicated above its respective graph. RNA levels were normalized using the geometric mean of the reference genes *GAPDH* and *UBC* and presented relative to control samples (*n* ≥ 3). Values are means ± SEM. ns, not significant; * *p* < 0.05. Abbreviation: HE, hypereosinophilic; lncRNA, long non-coding RNA; RNA, ribonucleic acid.

Following the *in silico* identification of eight promising lncRNA candidates associated with eosinophil-related disorders, we sought to validate their expression in human eosinophils isolated from the blood of healthy donors and HE patients—a condition characterized by a persistent AEC exceeding 1500 eosinophils/µL, while they account for less than 500 eosinophils/µL under normal conditions [9]. Interestingly, the lncRNA candidate *ITGB2-AS1* showed significant downregulation in blood eosinophils from HE patients compared with healthy donors (Figure 2A). Additionally, the lncRNA *RRN3P2* appeared to exhibit a similar trend, though the difference between HE and healthy eosinophils was not statistically significant (Figure 2B). In contrast, the other six lncRNAs—*AL109809.1*, *LINC01146*, *MIR210HG*, *PTPRN2-AS1*, *LINC00298*, and *LINC02285* (Figure 2C–H)—displayed an opposing trend, showing slight upregulation in HE blood eosinophils compared with healthy ones, although these differences remained statistically insignificant. Based on these findings, we decided to concentrate on further investigating the lncRNA candidate *ITGB2-AS1* in the subsequent experiments of this study.

### 3.3. Impaired Differentiation of Eosinophils with Reduced Expression of the lncRNA Candidate ITGB2-AS1

**Figure 3:**
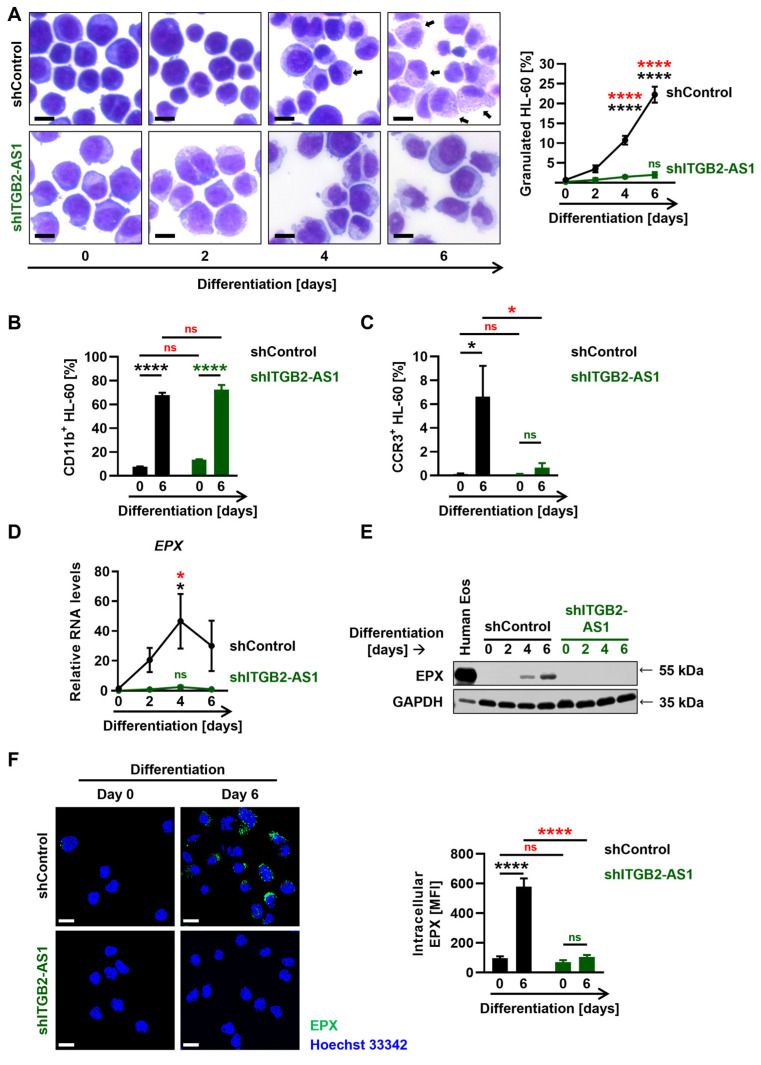
The effect of *ITGB2-AS1* lncRNA deficiency on eosinophil differentiation, granulogenesis, and EPX expression. (**A**–**F**) HL-60c15 cells were differentiated into ELCs in the presence of sodium butyrate and IL-5 for up to 6 days. (**A**) Cell morphology. (**Left**) Representative images of differentiating HL-60c15 following Hemacolor Rapid staining at the indicated days of differentiation. Images were acquired with the automatic digital slide scanner Pannoramic MIDI II. Intracellular granules are indicated by black arrows. Scale bars, 10 µm. (**Right**) The frequency of granulated cells was evaluated manually by light microscopy using a C plan 100×/1.25 Oil objective (*n* = 4). (**B**,**C**) Flow cytometry. The frequency of HL-60c15 cell differentiation was assessed by CD11b (**B**) and CCR3 (**C**) surface expression after exclusion of dead cells (*n* = 4). (**D**) Quantitative PCR. Relative RNA levels of the granule protein EPX in differentiating HL-60c15 cells after the indicated days of differentiation. *EPX* RNA levels were normalized using the geometric mean of the reference genes *GAPDH*, *UBC*, and *HPRT1* and presented relative to shControl cells at day 0 of differentiation (*n* ≥ 3). (**E**) Immunoblotting. Protein lysates were obtained from differentiating HL-60c15 cells at the indicated days of differentiation. EPX was detected using a monoclonal mouse anti-EPX antibody. GAPDH protein levels served as loading controls. Lysates from human blood eosinophils (Human Eos) were used as a positive control for the presence of EPX. A representative immunoblot of three independent experiments is shown. (**F**) Confocal microscopy. Differentiating HL-60c15 cells were stained for the eosinophil granule protein EPX and the nuclei using monoclonal mouse anti-EPX antibody and Hoechst 33342, respectively. (**Left**) Representative images of the presence of EPX in HL-60c15 cells at the indicated days of differentiation. (**Right**) Quantification of the mean fluorescence intensity (MFI) of intracellular EPX. Cells were delimited using “Surfaces” mode in Imaris, followed by EPX (green channel) MFI quantification (*n* = 4, with ≥42 cells per condition). Scale bars, 10 µm. Values are means ± SEM. ns, not significant; * *p* < 0.05. **** *p* < 0.0001. Significances in black illustrate the significance of shControl cells compared with undifferentiated (day 0) shControl cells. Significances in green denote the significance of shITGB2-AS1 cells compared with shITGB2-AS1 cells at day 0. Significances in red illustrate the significant difference between the shControl and shITGB2-AS1 cells. Abbreviations: ELC, eosinophil-like cell; EPX, eosinophil peroxidase; GAPDH, glyceraldehyde-3-phosphate dehydrogenase; HPRT1, hypoxanthine-guanine phosphoribosyltransferase 1; lncRNA, long non-coding RNA; kDa, kilodalton; MFI, mean fluorescence intensity; RNA, ribonucleic acid; UBC, ubiquitin C.

Considering the challenges associated with the genetic modification of human eosinophils and the inability to conduct functional screenings with mouse eosinophils due to the specificity of the lncRNA *ITGB2-AS1* to human cells, we employed the human promyelocytic leukemia cell line HL-60 clone 15 (HL-60c15, ATCC CRL-1964) for genetic modifications and subsequent functional studies [35,46]. To elucidate the role of the lncRNA candidate *ITGB2-AS1* in eosinophil biology, we conducted lentiviral transduction of HL-60c15 cells with constructs encoding shRNA targeting *ITGB2-AS1* (shITGB2-AS1) or a scrambled control (shControl). After stable transduction and puromycin selection, HL-60c15 cells were differentiated into eosinophil-like cells (ELCs) in the presence of 0.5 mM sodium butyrate and 10 ng/mL IL-5 [35,46]. A striking observation during differentiation was the absence of specific granules in shITGB2-AS1 cells compared with shControl cells, as assessed by light microscopy and quantification of granulated cells over the differentiation time-course of 6 days (Figure 3A). Furthermore, while forward scatter (FSC) measurements, indicative of relative cell size, did not show significant differences between the two groups in flow cytometry analyses (Figure S1A), the marked change in granularity was further reflected in the side scatter (SSC) measurements, which revealed increased cellular complexity in shControl cells compared with shITGB2-AS1 cells after 6 days of differentiation (Figure S1B).

To further evaluate the differentiation status of the cells, we examined the surface protein levels of the myeloid differentiation marker CD11b and the eosinophil differentiation marker CCR3. As anticipated, undifferentiated cells did not exhibit CD11b protein expression, whereas about 75% of the cells were positive for this marker after 6 days of differentiation (Figure 3B). Notably, there was no difference in the frequency of CD11b-positive cells between shITGB2-AS1 cells and shControl cells (Figure 3B). Moreover, while undifferentiated cells lacked CCR3 protein expression, shControl cells exhibited a significant increase in CCR3 expression after 6 days of differentiation, which was entirely absent in shITGB2-AS1 cells (Figure 3C). These data suggest a relevant role of the lncRNA *ITGB2-AS1* in eosinophil differentiation.

Given the absence of granules in shITGB2-AS1 cells, we investigated the expression of the granule protein EPX. In differentiating shControl cells, *EPX* mRNA levels were significantly upregulated, reaching maximal expression after four days of differentiation, whereas shITGB2-AS1 cells exhibited no *EPX* expression at the RNA level (Figure 3D). These findings were corroborated by immunoblot analysis, showing an increase in EPX protein levels after four days of differentiation, peaking at 6 days (Figure 3E). Human circulating blood eosinophils (Human Eos) served as a positive control (Figure 3E). In contrast, shITGB2-AS1 cells did not express EPX at the protein level. These results were further supported by confocal microscopy, showing strong EPX (Figure 3F) and MBP-1 (Figure S1C) protein levels in shControl cells after 6 days of differentiation, both of which were markedly reduced in shITGB2-AS1 cells.

We next investigated the effect of lncRNA *ITGB2-AS1* deficiency on cell proliferation. Interestingly, shITGB2-AS1 cells did not show increased proliferation compared with shControl cells, as measured by the absolute cell count in culture over 6 days of differentiation (Figure S1D) and Ki-67 protein levels (Figure S1E).

Taken together, our findings highlight the crucial role of lncRNA *ITGB2-AS1* in eosinophil differentiation, characterized by a marked reduction in cytoplasmic specific granules and decreased expression of the surface marker CCR3 and the eosinophil granule proteins EPX and MBP-1.

### 3.4. Several Eosinophil-Related Proteins Identified in the Correlation Network Analysis Are Downregulated in ITGB2-AS1-Deficient Cells

After revealing the role of *ITGB2-AS1* in eosinophil differentiation, we examined the network of eosinophil-related protein-coding genes whose mRNA expression was correlated with the lncRNA *ITGB2-AS1* (Figure 1B). This investigation aimed to determine whether *ITGB2-AS1* deficiency in the cells would lead to the downregulation of these proteins. We first sought to explore surface proteins by flow cytometry.

**Figure 4:**
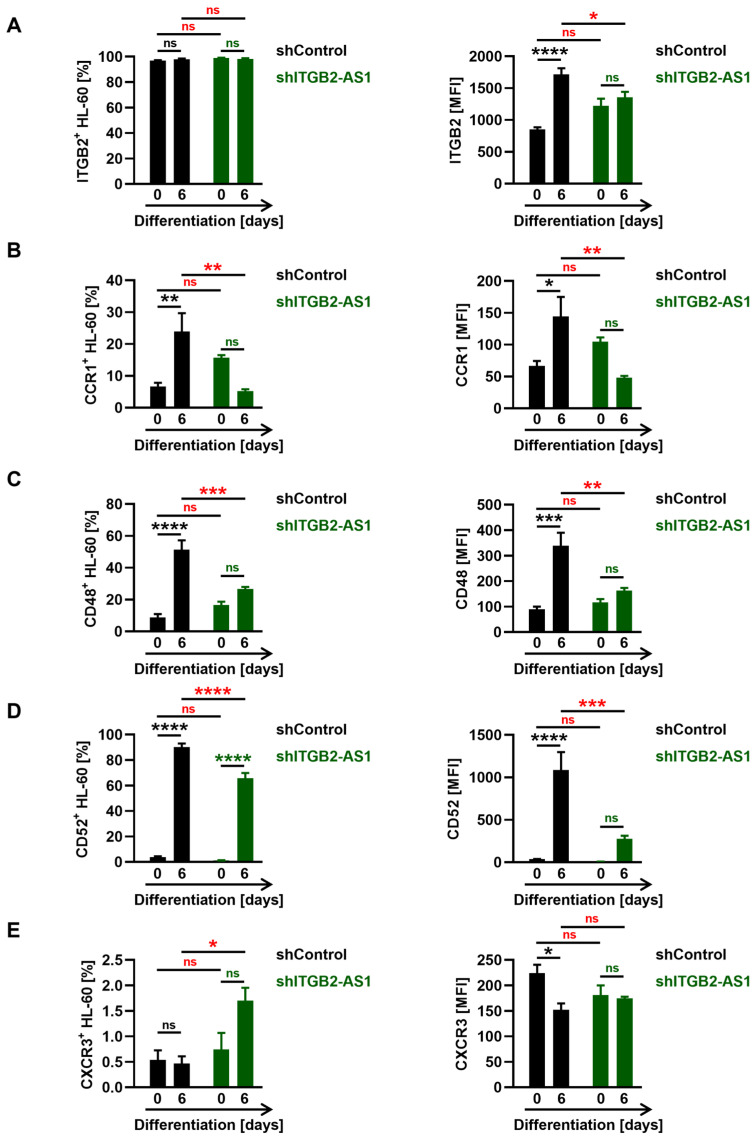
Expression of eosinophil-related proteins shown to be co-expressed with the lncRNA *ITGB2-AS1* in the correlation network analysis. (**A**–**E**) Flow cytometry. HL-60c15 cells were differentiated into ELCs in the presence of sodium butyrate and IL-5 for up to 6 days. The surface protein expression of ITGB2 (**A**), CCR1 (**B**), CD48 (**C**), CD52 (**D**), and CXCR3 (**E**) was assessed after the exclusion of dead cells (*n* ≥ 3). (**A**–**E**) (**Left**) Frequency of live cells expressing the proteins at the plasma membrane. (**Right**) Surface protein expression levels are represented as MFI in live cells. Values are means ± SEM. ns, not significant; * *p* < 0.05. ** *p* < 0.01; *** *p* < 0.001; **** *p* < 0.0001. Significances in black illustrate the significance of shControl cells compared with undifferentiated (day 0) shControl cells. Significances in green denote the significance of shITGB2-AS1 cells compared with undifferentiated shITGB2-AS1 cells. Significances in red illustrate a significant difference between the shControl and shITGB2-AS1 cells. Abbreviations: CXCR3, C-X-C motif chemokine receptor 3; ELC, eosinophil-like cell; ITGB2, integrin subunit beta 2; MFI, mean fluorescence intensity.

A previous study on breast cancer cells demonstrated that *ITGB2-AS1* expression could elevate ITGB2 (i.e., CD18) mRNA and protein levels [43], aligning with our correlation network from transcriptomic datasets of eosinophilic-related diseases (Figure 1B). Both undifferentiated and differentiated shControl and shITGB2-AS1 cells exhibited surface ITGB2 expression, as indicated by the frequency of ITGB2-positive cells (Figure 4A, left). Moreover, ITGB2 protein levels markedly increased from day 0 to day 6 of differentiation in shControl cells but not in shITGB2-AS1 cells, resulting in a significant difference in expression levels between the two cell types after 6 days of differentiation, as indicated by MFI (Figure 4A, right).

By examining additional surface markers, we observed a significant increase in CCR1 and CD48 protein levels in shControl cells after 6 days of differentiation compared with undifferentiated cells, both in the frequency of expressing cells and MFI levels (Figure 4B,C). Importantly, shITGB2-AS1 cells exhibited markedly lower surface protein levels of CCR1 and CD48 after 6 days of differentiation (Figure 4B,C). Additionally, CD52 expression followed a similar trend to CCR1 and CD48. Despite a significant increase of shITGB2-AS1 cells expressing CD52 after 6 days of differentiation (Figure 4D, left), the frequency of CD52-positive cells and MFI levels remained markedly lower in shITGB2-AS1 cells compared with shControl cells (Figure 4D).

Finally, the protein levels of the chemokine receptor CXCR3 were minimal, with less than 1% of shControl and 2% of shITGB2-AS1 cells exhibiting this marker after 6 days of differentiation (Figure 4E, left). However, there was no difference in CXCR3 MFI levels between the two cell types (Figure 4E, right).

Taken together, we identified a role for *ITGB2-AS1* not only in eosinophil differentiation but also in the expression of eosinophil-related proteins. This underscores the important role of *ITGB2-AS1* in eosinophil biology.

### 3.5. Eosinophils with a Deficiency in the lncRNA ITGB2-AS1 Exhibit Reduced Degranulation and Compromised Reactive Oxygen Species (ROS) Production

**Figure 5:**
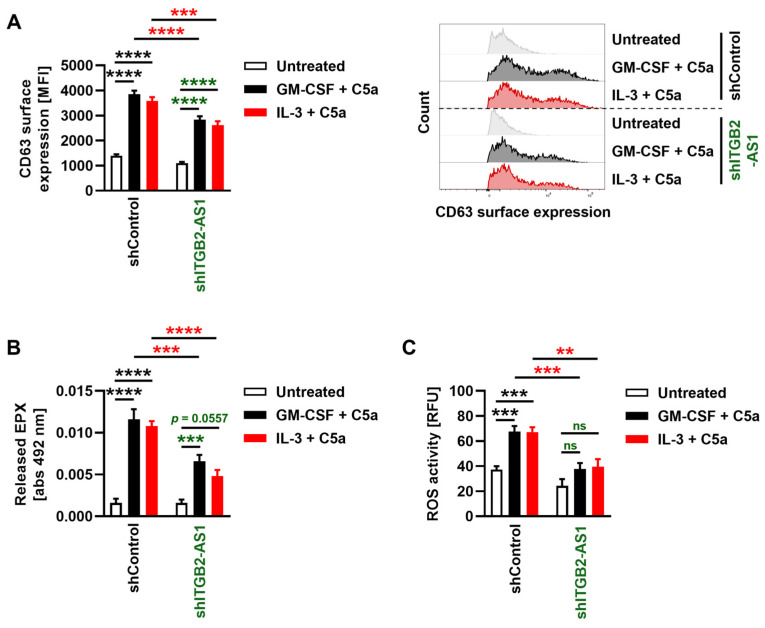
The impact of *ITGB2-AS1* lncRNA deficiency on eosinophil degranulation and ROS production. (**A**–**C**) HL-60c15 cells differentiated for 6 days in the presence of sodium butyrate and IL-5 were primed with GM-CSF or IL-3 for 20 min and subsequently stimulated with C5a for 30 min at 37 °C. (**A**,**B**) Degranulation assays. (**A**) Flow cytometry. Following the aforementioned stimulation, eosinophil degranulation was assessed by measuring CD63 surface expression (*n* = 5). (**Right**) A representative histogram of flow cytometry data is shown for each condition. (**B**) EPX assay. Subsequent to the previously mentioned stimulation, the release of the eosinophil granule protein EPX into the supernatant was evaluated by determining EPX activity using the peroxidase substrate O-phenylenediamine (OPD) and measuring absorbance at 492 nm (*n* = 5). (**C**) ROS production. Following the above-mentioned stimulation, ROS production was assessed by measuring DHR123 fluorescence with a spectrofluorometer (*n* = 8). Values are means ± SEM. ns, not significant; ** *p* < 0.01; *** *p* < 0.001; **** *p* < 0.0001. Significances in black illustrate the significance of shControl cells compared with untreated shControl cells. Significances in green denote the significance of shITGB2-AS1 cells compared with untreated shITGB2-AS1 cells. Significances in red illustrate the significant difference between the shControl and shITGB2-AS1 cells for the same condition. Abbreviations: abs, absorbance; C5a, complement component 5a; EPX, eosinophil peroxidase; GM-CSF, granulocyte-macrophage colony-stimulating factor; DHR123, dihydrorhodamine 123; IL-3, interleukin 3; IL-5, interleukin 5; ITGB2-AS1, ITGB2 antisense RNA 1; MFI, mean fluorescence intensity; OPD, O-phenylenediamine; RFU, relative fluorescence units; ROS, reactive oxygen species.

In addition to their more recently appreciated functions in tissue homeostasis, wound healing, and immunoregulation [4], eosinophils were traditionally viewed as cytotoxic effector cells due to their active role in host defense against diverse pathogens and their implication in the pathophysiology of inflammatory diseases [47]. Eosinophils employ a variety of extracellular mechanisms to defend against invading pathogens, including the generation of ROS, the release of toxic granule proteins through degranulation, and the formation of extracellular traps (EETs) [4]. Several studies have examined effector functions, such as degranulation, on neutrophil-like cells derived from the HL-60 cell line [48,49]. However, to our knowledge, no investigations have been conducted on the effector functions of ELCs derived from HL-60c15 cells. In the present study, we evaluated eosinophil degranulation by assessing the surface upregulation of the surrogate marker CD63 as well as the release of the granule protein EPX in the supernatant, as previously demonstrated in both human and murine eosinophils [33]. After 6 days of differentiation, both shControl and shITGB2-AS1 ELCs primed with GM-CSF or IL-3 and further stimulated with C5a showed significant upregulation of the degranulation marker CD63 compared with untreated cells (Figure 5A). Interestingly, *ITGB2-AS1*-deficient ELCs exhibited markedly reduced CD63 surface protein levels upon activation relative to shControl cells (Figure 5A). This reduction in degranulation was further supported by EPX release measurements, though IL-3-primed shITGB2-AS1 ELCs released less EPX compared with GM-CSF-primed ELCs (Figure 5B). Additionally, we demonstrated the inability of shITGB2-AS1 cells to produce ROS, as evidenced by the lack of an increase in ROS levels upon stimulation (Figure 5C). In contrast, stimulated shControl cells generated significantly higher ROS levels compared with both untreated cells and shITGB2-AS1 ELCs under identical stimulation conditions (Figure 5C). Collectively, these findings suggest that *ITGB2-AS1*-deficient ELCs have impaired degranulation and ROS production, indicating a key role for the lncRNA *ITGB2-AS1* in the regulation of both eosinophil maturation and eosinophil functions.

## 4. Discussion

Long non-coding RNAs (lncRNAs) are a heterogeneous class of extensive transcripts exceeding 200 nucleotides in length that do not encode proteins [16]. These molecules engage in a range of regulatory activities by interacting with DNA and RNA through base pairing and with proteins via their modular structures, thereby serving as scaffolds for proteins involved in specific biological processes [50]. LncRNAs were shown to influence gene expression by modulating transcription factor binding and by affecting the stability or translation rate of mRNAs, as well as the stability, activity, and localization of proteins [50,51]. In recent years, lncRNA-based therapeutics have attracted considerable attention, as these molecules may offer crucial insights into disease mechanisms and represent promising therapeutic targets and biomarkers for both diagnosis and prognosis [52,53,54]. Although lncRNA-specific therapeutics have not yet reached clinical translation, the growing number of approved RNA-based therapies (11 FDA/EMA-approved therapies to date) highlights the potential of this approach [55]. These advancements reinforce the potential of targeting lncRNAs, including *ITGB2-AS1*, in developing innovative treatments for eosinophil-related disorders.

Numerous studies investigated the role of lncRNAs in eosinophil-related diseases [18,19,20,21], primarily focusing on RNA expression levels in tissue homogenates. This approach has neglected the specific expression and functions of lncRNAs in eosinophils, leaving their potential role in the pathogenesis of related disorders insufficiently understood. In the current study, we identified 44 lncRNAs that were upregulated in blood eosinophils compared with other WBCs through a manual query of the Haemopedia Human RNA-Seq database [22]. This list was refined to eight lncRNAs whose expression correlated with eosinophil-related proteins in transcriptomic datasets of various eosinophilic disorders. Among these, we demonstrated that the lncRNA *ITGB2-AS1* was significantly downregulated in eosinophils isolated from the blood of patients with hypereosinophilia compared with healthy donors. Although *ITGB2-AS1* was the primary focus of this study, the remaining seven lncRNAs are also promising and should be explored in subsequent studies.

To further elucidate the role of *ITGB2-AS1* in eosinophil biology, we established a stable knockdown in the HL-60c15 cell line. Our findings revealed that *ITGB2-AS1*-deficient HL-60 cells exhibited impaired eosinophil differentiation, as indicated by a significant reduction in cytoplasmic specific granules and decreased expression of eosinophil granule proteins EPX and MBP-1. Additionally, after 6 days of differentiation, the surface expression of CCR3 was significantly downregulated in shITGB2-AS1 cells compared with shControl cells, while CD11b was not affected. It is noteworthy that CCR3, which is progressively expressed during eosinophil maturation, peaks in fully mature eosinophils, while CD11b is already highly expressed at the myelocyte stage, with both markers absent in promyelocytes [56]. This is consistent with our observations and suggests that the lncRNA *ITGB2-AS1* plays a crucial role in the transition from myelocyte to mature eosinophils, with *ITGB2-AS1*-deficient cells being arrested at the myelocyte stage.

Besides CCR3, shITGB2-AS1 cells displayed a marked reduction in the protein levels of the CC-chemokine receptor CCR1, a CC-chemokine receptor involved in eosinophil activation and migration [57]. Notably, a previous study demonstrated CCR1 to be expressed before and at higher levels than CCR3 in differentiating HL-60c15 cells [58].

Other surface markers, such as CD48 and CD52, were also found to be downregulated in shITGB2-AS1 cells following 6 days of differentiation. CD48 expression was previously found to be elevated on skin eosinophils from AD patients and in the presence of *Staphylococcus aureus* exotoxins but reduced in blood eosinophils from AD patients [59]. Moreover, eosinophils from nasal polyps and asthmatic patients’ blood also showed increased CD48 expression [60], while another study reported elevated CD48 expression in moderate asthma and reduced levels in severe asthma [61]. Additionally, CD52, found to be expressed on multiple cell types, including eosinophils [62], was shown to serve as a therapeutic target in severe or refractory cases of HESs [63], as well as for patients with relapsed and refractory erythrodermic cutaneous T cell lymphoma—a condition frequently associated with skin and peripheral blood eosinophilia [64]. Finally, ITGB2 (i.e., CD18) is a common β-subunit of the leukocyte integrin family of adhesion molecules and was shown to take part in direct eosinophil interaction with lymphocytes [65], but also airway epithelial cells [66], and to be involved in chemokine-mediated eosinophil tissue infiltration [67]. ITGB2 expression was reported to rely on the lncRNA *ITGB2-AS1* in breast cancer cells [43], a positive relationship that was also observed in the serum of patients with rheumatoid arthritis [68], as well as in our correlation network based on transcriptomic datasets of eosinophil-related conditions. Notably, the downregulation of several proteins, including ITGB2, CCR1, CD48, and CD52, whose expression correlated with the expression of the lncRNA *ITGB2-AS1* in our correlation analysis, further indicates effective knockdown. Interestingly, GM-CSF-enhanced adhesion and ROS production from *N*-formylmethionyl-leucyl-phenylalanine (fMLP)-stimulated eosinophils were inhibited when the cells were pre-treated with anti-CD18 antibodies [69], which is consistent with our findings, demonstrating a downregulation of ITGB2 expression in shITGB2-AS1 cells as well as impaired ROS production. In addition to impaired ROS production, shITGB2-AS1 cells exhibited reduced degranulation, indicating a broad impact of *ITGB2-AS1* deficiency on eosinophil differentiation and function. The observed effects on eosinophil function are likely partially due to impaired differentiation. Therefore, it would be valuable to further investigate the role of *ITGB2-AS1* by examining the effects of its downregulation in already differentiated cells, potentially using an inducible knockdown strategy, as well as assessing the consequences of its overexpression.

Regarding the limitations of this study, most experiments were conducted on the human promyelocytic leukemia cell line HL-60c15. Therefore, further investigation of the lncRNA *ITGB2-AS1* in human eosinophils is warranted, given its potential as a new therapeutic target for the treatment of eosinophil-related disorders. Finally, the regulatory mechanisms governing the expression of CCR1, CD18, CD48, and CD52 by *ITGB2-AS1* remain undetermined and require further investigation, with a suggested emphasis on the potential direct interaction between *ITGB2-AS1* and established transcriptional regulators of eosinophil differentiation.

## 5. Conclusions

In summary, we identify the lncRNA *ITGB2-AS1* as a critical regulator of eosinophil differentiation, evidenced by a reduction in specific cytoplasmic granules and downregulation of key granule proteins (EPX, MBP-1), chemokine receptors (CCR1, CCR3), and the integrin beta chain CD18, along with other eosinophil-related markers such as CD48 and CD52. *ITGB2-AS1* deficiency also impairs essential eosinophil functions, including degranulation and ROS production, positioning it as a promising therapeutic target for eosinophil-related disorders pending further *in vivo* validation.

## Figures and Tables

**Table 1:** List of reagents with supplier details.

Reagents	Supplier (Distributor)	Location
2-propanol	Merck Millipore	Darmstadt, Germany
Alexa Fluor^®^ 488 anti-human CD183 (CXCR3) (clone 1C6)	BD Biosciences	Allschwil, Switzerland
Alexa Fluor^®^ 488 anti-human Ki-67 (clone B56)	BD Biosciences	Allschwil, Switzerland
Alexa Fluor^®^ 647 anti-human CD191 (CCR1) (clone 53504)	BD Biosciences	Allschwil, Switzerland
APC anti-mouse/human CD11b (clone M1/70)	BioLegend	London, UK
BD Horizon™ Fixable Viability Stain 620	BD Biosciences	Allschwil, Switzerland
Black, glass-bottom 96-well plates	Greiner Bio-One	Frickenhausen, Germany
Bovine serum albumin (BSA)	Sigma-Aldrich	Buchs, Switzerland
Brilliant Violet 421™ anti-human CD193 (CCR3) (clone 5E8)	BioLegend	London, UK
Brilliant Violet 421™ anti-human CD48 (clone TU145)	BD Biosciences	Allschwil, Switzerland
Chloroform	Sigma-Aldrich	Buchs, Switzerland
Cytochalasin B	Sigma-Aldrich	Buchs, Switzerland
Dihydrorhodamine 123 (DHR123)	Sigma-Aldrich	Buchs, Switzerland
Diisopropyl fluorophosphate (DFP)	Sigma-Aldrich	Buchs, Switzerland
Dithiothreitol (DTT) solution	Sigma-Aldrich	Buchs, Switzerland
Dulbecco’s Phosphate-Buffered Saline (PBS)	Sigma-Aldrich	Buchs, Switzerland
EasySep Human Eosinophil Isolation Kit	StemCell Technologies	Cologne, Germany
EDTA (pH 8.0)	ThermoFisher Scientific (distributed by LuBioScience)	Lucerne, Switzerland
Ethanol	Merck Millipore	Darmstadt, Germany
Fetal calf serum (FCS)	GE Healthcare Life Sciences	Little Chalfont, UK
Glycerol	Sigma-Aldrich	Buchs, Switzerland
Glycogen	Invitrogen	Carlsbad, CA, USA
Hemacolor Rapid staining kit	Merck Millipore	Darmstadt, Germany
Hexadimethrine bromide (polybrene)	Sigma-Aldrich	Buchs, Switzerland
Hoechst 33342	ThermoFisher Scientific (distributed by LuBioScience)	Lucerne, Switzerland
HRP-conjugated secondary antibodies	GE Healthcare Life Sciences	Little Chalfont, UK
Human complement factor 5a (C5a)	Hycult Biotech	Uden, The Netherlands
Human GM-CSF	Novartis Pharma	Nuremberg, Germany
Human IL-3	R & D Systems	Abingdon, UK
Human IL-5	R & D Systems	Abingdon, UK
Hydrogen peroxide (H_2_O_2_) solution	Sigma-Aldrich	Buchs, Switzerland
Immobilon Forte Western HRP substrate	Merck Millipore	Darmstadt, Germany
iScript™ gDNA Clear cDNA Synthesis kit	Bio-Rad Laboratories	Cressier, Switzerland
iTaq Universal SYBR Green Supermix	Bio-Rad Laboratories	Cressier, Switzerland
Magnesium chloride (MgCl2) solution	Sigma-Aldrich	Buchs, Switzerland
Monoclonal mouse anti-human GAPDH antibody (clone 6C5)	Merck Millipore	Darmstadt, Germany
Monoclonal mouse anti-PRG2 (clone BMK13)	Abcam	Cambridge, MA, USA
Nonidet^®^ P-40 substitute	Fluka Biochemika	Buchs, Switzerland
Normal goat sera	Vector Laboratories	Burlingame, CA, USA
O-phenylenediamine dihydrochloride (OPD)	Sigma-Aldrich	Buchs, Switzerland
Pancoll Human	PAN-Biotech	Aidenbach, Germany
Paraformaldehyde (PFA) 16% Aqueous Solution EM Grade	Electron Microscopy Sciences (distributed by Lucerna-Chem AG)	Lucerne, Switzerland
PE anti-human CD18 (ITGB2) (clone 6.7)	BD Biosciences	Allschwil, Switzerland
PE anti-human CD52 (clone 4C8)	BD Biosciences	Allschwil, Switzerland
Phenylmethylsulfonyl fluoride (PMSF)	Sigma-Aldrich	Buchs, Switzerland
PhosSTOP™ phosphatase inhibitor cocktail	Roche Diagnostics	Rotkreuz, Switzerland
Pierce BCA protein assay kit	ThermoFisher Scientific (distributed by LuBioScience)	Lucerne, Switzerland
Polyvalent human IgG	Gift from CSL Behring	Bern, Switzerland
Potassium bicarbonate (KHCO_3_)	Sigma-Aldrich	Buchs, Switzerland
Primary monoclonal mouse anti-mouse EPX (clone MM25-82.2)	Lee Laboratories (Mayo Clinic)	Scottsdale, AZ, USA
Prolong Gold Antifade mounting medium	ThermoFisher Scientific (distributed by LuBioScience)	Lucerne, Switzerland
Protease inhibitor cocktail	Sigma-Aldrich	Buchs, Switzerland
Quick-RNA™ Microprep Kit	Zymo Research (distributed by Lucerna-Chem AG)	Lucerne, Switzerland
RNasin^®^ Plus Ribonuclease Inhibitor	Promega AG	Dübendorf, Switzerland
RPMI-1640/GlutaMAX medium	Sigma-Aldrich	Buchs, Switzerland
Saponin	Sigma-Aldrich	Buchs, Switzerland
Secondary antibody goat anti-mouse Alexa Fluor 488	ThermoFisher Scientific (distributed by LuBioScience)	Lucerne, Switzerland
Sodium chloride (NaCl) solution	Sigma-Aldrich	Buchs, Switzerland
Sodium deoxycholate monohydrate	Sigma-Aldrich	Buchs, Switzerland
Sodium orthovanadate (Na_3_VO_4_)	Sigma-Aldrich	Buchs, Switzerland
Sodium pyrophosphate	Sigma-Aldrich	Buchs, Switzerland
Triton X-100	Sigma-Aldrich	Buchs, Switzerland
Trizma^®^ hydrochloride solution pH 7.4	Sigma-Aldrich	Buchs, Switzerland
Trizma^®^ hydrochloride solution pH 8.0	Sigma-Aldrich	Buchs, Switzerland
Tween 20	Sigma-Aldrich	Buchs, Switzerland
X-tremeGENE™ HP DNA Transfection Reagent	Roche Diagnostics	Rotkreuz, Switzerland
X-VIVO 15 medium	Lonza	Walkersville, MD, USA

**Table 2:** List of transcriptomic datasets with accession numbers and sample origins.

Condition	Accession Number	Sample Origin
Allergic rhinitis	GSE101720	Nasal, bronchial swab
	GSE72713	Nasal swab
Bronchial asthma	GSE85214	Bronchial swab
	GSE117038/GSE106230	Whole blood
Eosinophilic esophagitis (EoE)	GSE41687	Esophageal biopsy
	GSE58640	Esophageal biopsy
	GSE148381	Esophageal biopsy
Atopic dermatitis (AD)	GSE121212	Skin biopsy
	GSE140380	Skin biopsy
Eosinophilic granulomatosis with polyangiitis (EGPA)	GSE144302	Lung biopsy

## Data Availability

All data presented in this study are available on request from the corresponding author.

## Supplementary Materials

The following supporting information can be downloaded at: https://www.mdpi.com/article/10.3390/cells13231936/s1, Figure S1: The impact of *ITGB2-AS1* lncRNA deficiency on eosinophil differentiation, MBP-1 expression, and proliferation.; Table S1: List of eosinophil-related protein-coding genes by category with references; Table S2: List of shRNAs obtained from OBiO Technology (Shanghai, China); Table S3: Primer sequences for quantitative RT-qPCR assays.

## Abbreviations

abs: absorbance; AD, atopic dermatitis; AEC, absolute eosinophil count; BANCR, BRAF-activated non-coding RNA; BSA, bovine serum albumin; C5a, complement factor 5a; cDNA, complementary DNA; CMP, common myeloid progenitor; CXCR3, C-X-C motif chemokine receptor 3; DC, dendritic cell; DFP, diisopropyl fluorophosphate; DHR 123, dihydrorhodamine 123; DNA, deoxyribonucleic acid; DTT, dithiothreitol; EDN, eosinophil-derived neurotoxin; EDTA, ethylenediaminetetraacetic acid; EET, eosinophil extracellular trap; EGPA, eosinophilic granulomatosis with polyangiitis; ELC, eosinophil-like cell; EoE, eosinophilic esophagitis; EoP, eosinophil precursor; EPX, eosinophil peroxidase; FCS, fetal calf serum; fMLP, N-formylmethionyl-leucyl-phenylalanine; FSC, forward scatter; GAPDH, glyceraldehyde-3-phosphate dehydrogenase; gDNA, genomic DNA; GM-CSF, granulocyte-macrophage colony-stimulating factor; HE, hypereosinophilia; HESs, hypereosinophilic syndromes; HL-60c15, HL-60 clone 15; HPRT1, hypoxanthine-guanine phosphoribosyltransferase 1; HRP, horseradish peroxidase; HSC, hematopoietic stem cell; IL, interleukin; ITGB2, integrin subunit beta 2; ITGB2-AS1, ITGB2 antisense RNA 1; kDa, kilodalton; lncRNA, long non-coding RNA; MB, myeloblast; MBP-1, major basic protein-1; MC, myelocyte; MFI, mean fluorescence intensity; NK, natural killer; OPD, O-phenylenediamine dihydrochloride; PBS, phosphate-buffered saline; PFA, paraformaldehyde; pMC, promyelocyte; PMSF, phenylmethylsulfonyl fluoride; RNA, ribonucleic acid; RNA-seq, RNA sequencing; ROS, reactive oxygen species; RT, room temperature; shRNA, short hairpin RNA; SSC, side scatter; TBST, Tris-buffered saline with Tween^®^ 20 Detergent; UBC, ubiquitin C; WBC, white blood cell.
