# Supplementary material for: Evidence for a Role of the Long Non-Coding RNA ITGB2-AS1 in Eosinophil Differentiation and Functions
Source: Cells. 2024 Nov 22;13(23):1936. doi: 10.3390/cells13231936 (PMC11640120; doi:10.3390/cells13231936)
Supplement: Supplementary file 1 [file cells-13-01936-s001.zip › cells-3320497-supplementary.pdf]

Supplementary Materials

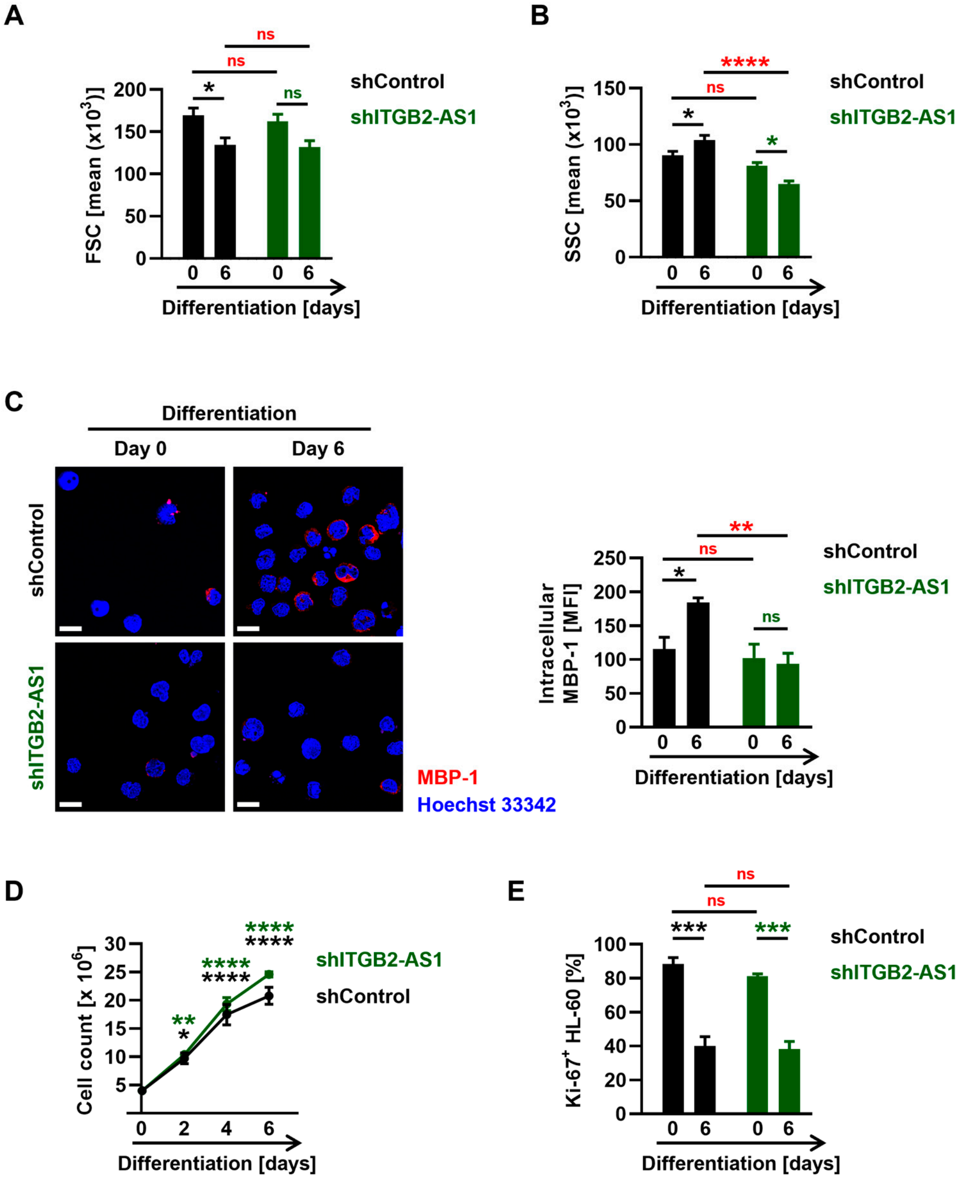

Figure S1. The impact of *ITGB2-AS1* lncRNA deficiency on eosinophil differentiation, MBP-1 expression, and proliferation. (**A**-**E**) HL-60c15 cells were differentiated into eosinophils in the presence of sodium butyrate and IL-5 for up to 6 days. (**A**,**B**) Flow cytometry. Cell morphology was assessed by measuring the mean of FSC (**A**) and SSC (**B**) (n = 7). (**C**) Confocal microscopy. Differentiating HL-60c15 cells were stained for the eosinophil granule protein MBP-1 and the nuclei using monoclonal mouse anti-PRG2 antibody and Hoechst 33342, respectively. (**Left**) Representative images of the presence of MBP-1 in HL-60c15 cells at the indicated days of differentiation. (**Right**) Quantification of MFI of intracellular EPX. Cells were delimited using "Surfaces" mode in Imaris followed by MBP-1 (red channel) MFI quantification (n = 4, with ≥ 41 cells per condition). Scale bars, 10 µm. (**D**,**E**) Cell proliferation. The proliferative status of differentiating HL-60c15 cells was evaluated by absolute cell count (**D**) at the indicated days of differentiation using an automated hematology analyzer (Sysmex Digitana), and by determining the frequency of cells expressing Ki-67 via flow cytometry (**E**) (n = 3). Values are means ± SEM. ns, not significant; * $p < 0.05$; ** $p < 0.01$; *** $p < 0.001$; **** $p < 0.0001$. Significances in black illustrate a significance of shControl cells compared to undifferentiated (day 0) shControl cells. Significances in green denote a significance of shITGB2-AS1 cells compared to undifferentiated shITGB2-AS1 cells. Significances in red illustrate a significant difference between the shControl and shITGB2-AS1 cells. Abbreviations: FSC, forward scatter; ITGB2-AS1, ITGB2 antisense RNA 1; MBP-1, major basic protein 1; MFI, mean fluorescence intensity; SSC, side scatter.

Table S1. List of eosinophil-related protein-coding genes by category with references.

**Regulators of eosinophil maturation** [23,24]	**Secretory Mediators** [27-29]	**Surface markers** [25,26]			**Granule and other proteins** [7,29]
CEBPA	*CCL11*	*ADAM8*	*CXCR4*	*IL5RA*	*CLC*
CEPBE	*CCL13*	*ADGRE5*	*CYSLTR1*	*IL9R*	*EPX*
GATA1	*CCL17*	*C3AR1*	*CYSLTR2*	*ITGA4*	*MMP9*
GATA2	*CCL2*	*CCR1*	*ENTPD1*	*ITGA6*	*PRG2*
GFI1	*CCL22*	*CCR2*	*F2RL1*	*ITGAD*	*RNASE2*
ID2	*CCL23*	*CCR3*	*FAS*	*ITGAL*	*RNASE3*
IL33	*CCL3*	*CD151*	*FCAR*	*ITGAM*	*TIMP1*
IL5	*CCL5*	*CD24*	*FCER1A*	*ITGAX*	
IRF8	*CSF2*	*CD28*	*FCER1G*	*ITGB1*	
KLF5	*CXCL1*	*CD33*	*FCGR2A*	*ITGB2*	
PU.1	*CXCL10*	*CD37*	*FCGR2B*	*ITGB7*	
RhoH	*CXCL5*	*CD4*	*FCGR2C*	*KIT*	
TRIB1	*CXCL8*	*CD44*	*FCGR3A*	*KLRB1*	
XBP1	*CXCL9*	*CD47*	*FCGR3B*	*LILRA2*	
ZFPM1	*IFNG*	*CD48*	*FPR1*	*LILRB1*	
	IL10	*CD52*	*FUT3*	*LILRB2*	
	IL11	*CD53*	*GPR135*	*LILRB3*	
	IL12A	*CD58*	*GSF2RB*	*LTB4R*	
	IL12B	*CD63*	*HLA-A*	*LTB4R2*	
	IL13	*CD69*	*HLA-B*	*PTGDR2*	
	IL16	*CD81*	*HLA-C*	*PTPRJ*	
	IL17A	*CD82*	*HLA-DRA*	*SELL*	
	IL1A	*CD86*	*HLA-DRB*	*SELPLG*	
	IL1B	*CD88*	*HRH1*	*SIGLEC10*	
	IL2	*CD9*	*HRH2*	*SIGLEC8*	
	IL25	*CD99*	*HRH4*	*SLC44A1*	
	IL3	*CEACAM1*	*ICAM1*	*SLC7A5*	
	IL4	*CEACAM5*	*IFNGR1*	*SPN*	
	IL5	*CEACAM6*	*IL13RA*	*TGFBR1*	
	IL6	*CEACAM8*	*IL2RA*	*TLR7*	
	IL9	*CR1*	*IL3R*	*TLR8*	
	KITLG	*CSF2RA*	*IL4R*	*TNFRSF1A*	
	NGF	*CXCR3*			
	PDGFB				
	TGFA				
	TGFB1				
	TNF				
	TNFSF13				
	VEGFA				

Table S2. List of shRNAs obtained from OBiO Technology (Shanghai, China).

shRNA	Targeted gene	gene ID	sequence
shITGB2-AS1	*ITGB2-AS1*	100505746	AGGCAGAAAGGGCACCCATTACTC<br>GAGTAATGGGTGCCCTTTCTGCCT
shControl	---	---	Scrambled<br>(exact sequence not provided)

Table S3. Primer sequences for quantitative RT-qPCR assays.

Target gene	Primer	Sequence
human *PTPRN2-AS1*	Forward:	5′-GAACAGACAGGACGTTTCACAG-3′
	Reverse:	5′-TTCTCAGGGCCTCAGCAG-3′
human *RRN3P2*	Forward:	5′-GGAAGACATGAGTGCTGAAGAG-3′
	Reverse:	5′-TGGTTGATGGAATACAGGAATGA-3′
human *MIR210HG*	Forward:	5′-CCTGGCATGGACTCGGAC-3′
	Reverse:	5′-GGACCCCAAGACCACACAC-3′
human *ITGB2-AS1*	Forward:	5′-GACCTGCTTCCATCAGATTCAC-3′
	Reverse:	5′-GTTCCTCCGCTCCATTCTCT-3′
human *AL1009809.1*	Forward:	5′-CCACTTCTATCCCGCCCAC-3′
	Reverse:	5′-GACCACCCAGCAAGCAAACTCA-3′
human *LINC00298*	Forward:	5′-AGTGCCAGTTTGAATGAAGGA-3′
	Reverse:	5′-CAATGTGTGATATTATGTGCAGAG-3′
human *LINC01146*	Forward:	5′-GCCCCACCTTCTGCTACTA-3′
	Reverse:	5′-TTATTCTTCCTTTCTTCTTCCTGC-3′
human *LINC02285*	Forward:	5′-CCCTCTGATGCTGAAGCCTTT-3′
	Reverse:	5′-GACTTGCACTGACTCCATGATGAT-3′
human *UBC*	Forward:	5′-CGGGATTTGGGTCGCAGT-3′
	Reverse:	5′-CGAAGATCTGCATTGTCAAGT-3′
human *GAPDH*	Forward:	5′-CAACAGCCTCAAGATCATCAGCAA-3′
	Reverse:	5′-CATGAGTCCTTCCACGATACC-3′
human *HPRT1*	Forward:	5′-ATGGTCAAGGTCGCAAG-3′
	Reverse:	5′-AAGTATTCATTATAGTCAAGGGCATATC-3′
human *EPX*	Forward:	5′-CTTTGGCCACACAATGCT-3′
	Reverse:	5′-TCCTCCTCACTTGCCGAAAC-3′
